# Proteomic Characterization of Cellular and Molecular Processes that Enable the *Nanoarchaeum equitans-Ignicoccus hospitalis* Relationship

**DOI:** 10.1371/journal.pone.0022942

**Published:** 2011-08-03

**Authors:** Richard J. Giannone, Harald Huber, Tatiana Karpinets, Thomas Heimerl, Ulf Küper, Reinhard Rachel, Martin Keller, Robert L. Hettich, Mircea Podar

**Affiliations:** 1 Chemical Sciences Division, Oak Ridge National Laboratory, Oak Ridge, Tennessee, United States of America; 2 Lehrstuhl für Mikrobiologie und Archaeenzentrum, Universität Regensburg, Regensburg, Germany; 3 Biosciences Division, Oak Ridge National Laboratory, Oak Ridge, Tennessee, United States of America; 4 Department of Microbiology, University of Tennessee, Knoxville, Tennessee, United States of America; Max-Planck-Institute for Terrestrial Microbiology, Germany

## Abstract

*Nanoarchaeum equitans*, the only cultured representative of the Nanoarchaeota, is dependent on direct physical contact with its host, the hyperthermophile *Ignicoccus hospitalis*. The molecular mechanisms that enable this relationship are unknown. Using whole-cell proteomics, differences in the relative abundance of >75% of predicted protein-coding genes from both Archaea were measured to identify the specific response of *I. hospitalis* to the presence of *N. equitans* on its surface. A purified *N. equitans* sample was also analyzed for evidence of interspecies protein transfer. The depth of cellular proteome coverage achieved here is amongst the highest reported for any organism. Based on changes in the proteome under the specific conditions of this study, *I. hospitalis* reacts to *N. equitans* by curtailing genetic information processing (replication, transcription) in lieu of intensifying its energetic, protein processing and cellular membrane functions. We found no evidence of significant *Ignicoccus* biosynthetic enzymes being transported to *N. equitans*. These results suggest that, under laboratory conditions, *N. equitans* diverts some of its host's metabolism and cell cycle control to compensate for its own metabolic shortcomings, thus appearing to be entirely dependent on small, transferable metabolites and energetic precursors from *I. hospitalis*.

## Introduction

The hyperthermophiles *Nanoarchaeum equitans* and *Ignicoccus hospitalis* engage in the only physical and specific cell-cell interaction documented thus far for two species of Archaea [1]. With a highly reduced genome and nearly absent biosynthetic and energetic functions, *N. equitans* requires physical contact with *I. hospitalis* [1], [2]. This enables uptake of small molecules (amino acids, lipids) from the host by some yet unknown mechanisms [2], [3]. Categorizing the interaction between the two organisms to a specific type of traditionally defined symbiotic relationship has been difficult due to the unclear impact that *N. equitans* has on its host which, in laboratory cultures, ranges from neutral to inhibitory [2]. Evidence of horizontal gene transfer between the two organisms and indication of genome streamlining in *I. hospitalis* nevertheless suggests that, at the genome level, the interaction has impacted not only *N. equitans* but its host as well [4]. As it represents the simplest “symbiotic” system known thus far, functional analysis of the roughly 1800 genes combined between the two organisms provides unique insight into specific genetic and physiological mechanisms of their relationship and can perhaps address more general principles of microbial interspecies interaction and co-evolution.

Among the approaches that enable global monitoring of genome function, whole-cell shotgun proteomics provides direct protein measurements and thus physical evidence for the expression of any individual protein-encoding gene from the genome [5]. When relative protein abundance levels are determined, quantitative variations of individual proteins can be linked to physiological activity or other measurable phenotypic states of the cell. Previous studies, which focused on identifying major proteins expressed by *I. hospitalis* grown as a pure culture [6] or those found in membrane fractions prepared from co-culture with *N. equitans* [7] detected a limited subset of the predicted proteome of the two organisms. For other archaeal systems, differential proteomics has been successfully applied to identify genes/proteins that are involved in adaptation and physiological response to various environmental challenges such as low temperature in *Methanococcoides burtonii* [8] or energy and nutrient starvation in *Methanococcus maripaludis* [9] and *Halobacterium salinarum* [10]. In this study, we applied an on-line, two-dimensional liquid chromatography tandem mass spectrometry (2D-LC-MS/MS) proteomic approach, utilizing the high resolving power and accuracy of a hybrid LTQ/Orbitrap mass spectrometer to maximize the number of detected proteins in several conditional whole-cell lysates. Normalized spectral abundance factors were employed to quantitatively compare the proteomic differences between *I. hospitalis* grown by itself or when engaged with *N. equitans*. Proteins that exhibit changes in their abundance levels per condition were identified, leading to a better understanding of the impact that *N. equitans* has on its host, both in terms of changes in gene expression and/or protein abundance thus providing physiological insight into the nature of their relationship.

## Results and Discussion

### Proteome coverage overview

Deep whole-cell proteomic coverage for *I. hospitalis* and *N. equitans* was achieved using Multidimensional Protein Information Technology (MudPIT) 2D-LC-MS/MS [11]. More importantly, the relative abundance of detected proteins was determined and compared between both *I. hospitalis* grown independently and in conjunction with *N. equitans*. By comparing *I. hospitalis* protein abundance between states, potential genes, proteins or cellular processes that respond to and may be involved in the association of these two archaea could be identified.

To compare the protein abundance profiles of both sets of samples (*I. hospitalis* and *I. hospitalis*-*N. equitans* co-culture), separate large-scale fermentations were performed. As previously shown, the number of *N. equitans* cells that are associated with any given host cell increases during the course of cultivation, from an average of less than one in early growth stages to over ten towards stationary phase [2]. While it is understood that the potential physiological differences associated with co-culture progression would be better defined by replicate time-course samples, this study focused more on developing a whole cell proteomic approach suitable for studying these organisms and thus used a single, stationary culture time point as the basis for comparison. In an effort to be comprehensive, an *N. equitans* sample was purified from a co-culture and analyzed by 2D LC-MS/MS, though quantitative analysis was not performed as this sample was derived from a co-culture with *I. hospitalis* and is thus not biologically relevant with regard to differential proteomics.

Three replicate measurements were obtained for each of the samples totaling nine, independent 2D LC-MS/MS runs. Average false-discovery rates were ascertained to be 2.19%, 2.76%, and 4.54% for the co-culture, *I. hospitalis* pure culture, and purified *N. equitans* samples respectively. Global pair-wise reproducibility between technical replicates at individual protein level ranged from 0.90–0.99 with average correlation values of 95.4% and 94.7% for *I. hospitalis* proteins or 93.4% and 92.2% for *N. equitans* proteins, in the co-culture or isolates respectively. Correlation values between the pure and co-cultures calculated independently for each organism, i.e. *I. hospitalis* proteins in the single vs. co-culture (86.3%) or *N. equitans* proteins in the purified sample vs. co-culture (93.2%) indicate the extent to which each organism's proteome varies as a function of culture condition. As follows, *I. hospitalis'* proteome is more dynamic than that of *N. equitans* and shows a marked correlational difference between technical replicates and culture condition. In contrast, the variability between *N. equitans* technical replicates is very similar to that observed between samples, suggesting little difference between the proteomes of each state. As a “pure” *N. equitans* sample can only be derived from co-culture with *I. hospitalis*, the proteomes were expected to be similar between conditions. Thus, the ensuing differential proteomic analysis focused on *I. hospitalis* samples. Further details about the data analysis strategy are provided online (**Supporting information S1 and Figure S1**).

Overall, a total of 1058 *I. hospitalis* proteins were identified out of 1444 open reading frames (ORFs), representing over 73% of the predicted proteome (**Table S1**). This remarkable level of proteome coverage suggests that a larger proportion of the proteome is constitutively expressed and supports the hypothesis that the *I. hospitalis* genome is streamlined, containing few redundant or nonfunctional genes [4]. The total number of SEQUEST-assigned spectra was similar between all sample conditions: 139,556 for *I. hospitalis* when grown as a pure culture, 145,894 assigned to the co-culture (68.5% to *I. hospitalis* and 31.5% to *N. equitans*), and 133,418 assigned to the purified *N. equitans* sample (90.0% to *N. equitans* and 10.0% to *I. hospitalis*). Across both *N. equitans* datasets (purified plus co-culture), we detected a total of 476 proteins out of the predicted 556 (85%). This is amongst the highest proteome coverage ever reported for an organism [12].

**Figure 1:**
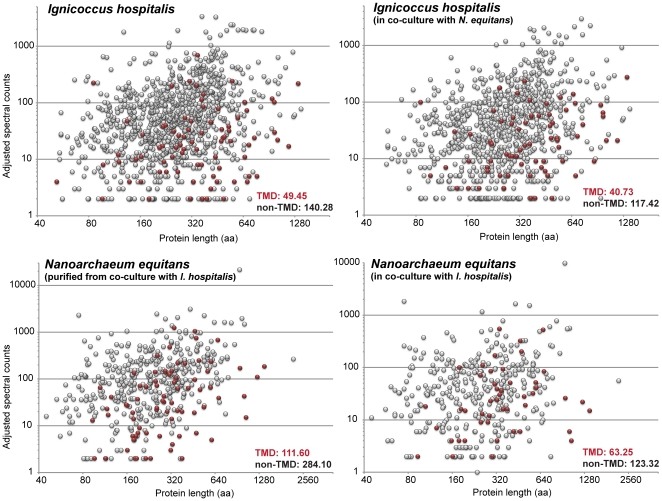
Analysis of protein size and membrane association effects on the number of assigned spectra. Each circle represents an individual detected protein. Each panel represents an individual sample. Proteins with predicted transmembrane domains (TMD) are in red. The average adjusted spectral counts for proteins containing TMDs or not were calculated for each of the four biological samples. There is no correlation between protein size and the number of detected spectra, however, membrane proteins generated on average approximately three times less spectra than non-TMD proteins.

Individual, pre-normalized protein spectra for each organism covered over three orders of magnitude per MS run, from 2 spectra (our conservatively defined minimum) to over 8000. At the whole proteome level, no correlation was observed between the size of a protein and the number of spectra obtained for that protein, especially after NSAF-based normalization, which effectively corrects for any protein length-derived abundance bias (**Figure 1**). When analyzing proteins containing predicted transmembrane domains (TMD), however, we observed a 2–3 fold lower average of the number of spectra per protein as compared to non-membrane proteins; these SpC values were independent of size as well (**Figure 1**). The general protocol for whole-cell shotgun proteomics is known to be superior for soluble proteins relative to membrane proteins, and thus translated into a lower fraction of identified transmembrane proteins as compared to total proteins (48.2% for both *Ignicoccus* and *Nanoarchaeum* combined compared to 82.5% of cytosolic proteins).

### Overview of detected *I. hospitalis* and *N. equitans* protein families

**Figure 2:**
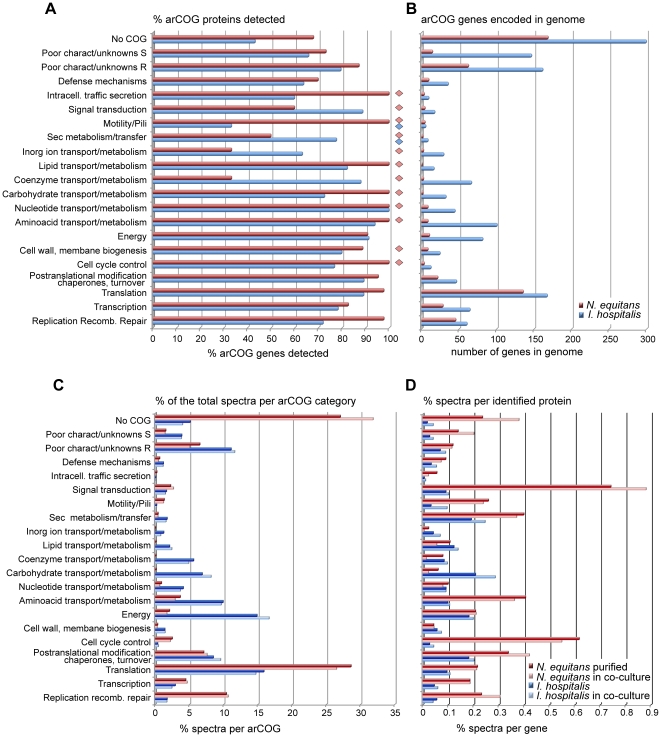
Proteomic coverage of *I. hospitalis* and *N. equitans* functional gene categories (arCOGs). (A) Summary of arCOG proteins detection (% of total for each individual arCOG class). The values represent the combined samples (single culture/purified and co-culture) for *I. hospitalis* (blue) and *N. equitans* (red). The diamonds indicate classes with low representation in the genome (<10 predicted proteins). (B) Number of predicted genes/proteins assigned to the arCOG classes in the two genomes. (C) Percentage of identified spectra assigned to the individual arCOG classes relative to the total proteome from each of the four samples. (D) Average spectral representation assigned to individual proteins depending on the arCOG class (% of total spectra).

Based on comparative genomic analyses, archaeal proteins have been assigned to orthologous groups (archaeal COGs, arCOGs) [13]. They reflect diversification of gene families and allow functional inferences to be made for individual organisms based on gene acquisition (duplication, horizontal transfer) or loss. In both *N. equitans* and *I. hospitalis*, arCOG-based analyses have revealed a low degree of functional redundancy (paralogs), gene loss and a high fraction of hypothetical genes with no homologs in other genomes (300 genes, ∼20% of the genome in *I. hospitalis*, and 169 genes, 30% of the genome in *N. equitans*) [4]. Though denoted as “hypothetical” as gene calls, approximately half of their encoded proteins were detected in the analyzed samples, indicating that they are valid and expressed genes that should be reclassified as “proteins with unknown function”. Among the arCOG categories, as expected, a large fraction (70–90%) of the proteins expressed in both organisms is involved in cell cycle control and genetic information processing (DNA replication and repair, transcription, translation and protein processing (**Figure 2**). Notably, we detected over 90% of the *I. hospitalis* proteins predicted to be involved in energy generation, amino acid and nucleotide metabolism and transport, again supporting the notion that this Archaeon has a streamlined, non-redundant genome in which most metabolic genes are constitutively expressed, even in laboratory setting. Functional categories that were covered to a lesser degree include genes with no assigned orthology to other genomes (40–50% of the predicted genes in that group) and/or cellular functions that are represented by a relatively low number of genes which may or may not respond to environmental variables not reproduced in the laboratory (inorganic ion transport, defense, motility/pili). A large number of genes in those categories also encode membrane proteins, which are in general less effectively detected by trypsin-based bottom-up proteomic methodologies and are therefore under-represented with regards to protein identification and/or overall sequence coverage. Even in those categories, however, several functions were clearly active, with corresponding proteins detected at significant levels (RSpC values 100–500). For example, the protein encoded by Iho_0670, which has been shown to form a unique type of cell surface appendage/fiber [14], was present in appreciable amounts in both single culture and co-culture with *N. equitans*.

In *N. equitans*, arCOG categories that encode nearly all primary metabolic functions are severely underrepresented, containing ten or fewer genes per category [15] (**Figure 2b**). Although this small subset of enzymes cannot support independent metabolism, detection of a large portion of these proteins (between 70–100%) indicates that *N. equitans* has not entirely lost its physiological capabilities. Even though overall the contribution of the metabolic COG classes to the total *N. equitans* proteome is modest, when normalized to the actual number of genes they include the normalized abundance of those expressed proteins is significant (**Figure 2c,d**). Interestingly, several enzymes present at significant levels are likely involved in amino acid and nucleotide metabolism (deaminases, dehydrogenases, kinases and hydrolases) and may perform a limited but important range of molecular inter-conversions and/or metabolic transformations of precursors imported from *I. hospitalis*, or perhaps recycling of its own metabolic products. An abundant inorganic pyrophosphatase (Neq461) prevents accumulation of pyrophosphate from DNA and RNA synthesis. The ATP synthase subunit A (Neq103) and the very short subunit B (Neq263) are expressed at appreciable and relatively equivalent levels. Likewise, all the other predicted subunits were detected except for the transmembrane oligomeric subunit c (NEQ217). The ATP-biosynthetic capability of the complex has not yet been experimentally confirmed as it lacks several subunits [16]; therefore, the energetic independence of *N. equitans* remains questionable. Aside from ATP synthase subunits, several redox proteins were also detected, including a predicted ferredoxin (Neq373), flavodoxin reductase (Neq051) and desulfoferredoxin (Neq011). The genetic information processing categories were covered extensively, with near 100% of the predicted proteins being detected. The full list of *I. hospitalis* and *N. equitans* expressed genes classified by functional arCOG categories and their relative protein abundance is presented in the **Table S1**.

### The *I. hospitalis* proteome

Since spectral abundance is a relative quantification measure, proteins for which a large number of spectra were detected indicate major cellular constituents. By identifying such highly abundant proteins, important inferences about cellular processes and/or structures may be drawn. However, as mentioned above, quantification of membrane and other smaller proteins is likely underestimated by virtue of the relative lack of tryptic cleavage sites leading to MS-incompatible peptides. Therefore, the proteome map of *I. hospitalis-N. equitans* is still somewhat incomplete, despite that over 75% of the joint, predicted proteome of the two constituent Archaea was identified with relatively conservative criteria. This value ranks with some of the most thoroughly identified proteomes to date, most notably representatives of the genus *Mycoplasma* [12].

Inferences into cellular function are perhaps more easily understood by observing abundance trends in proteins that belong to similar or linked functional categories. One of the protein categories present in high abundance in *I. hospitalis* includes chaperones and proteins involved in the oxidative stress response (**Table 1**). These include peroxiredoxin (Iho0459), an Hsp20 family protein (Iho_1363), and the thermosome subunits (Iho_0096 and 0897). The thermosome is a type of chaperone involved in protein folding and molecular adaptation to high temperature and has been previously identified as a major cytosolic protein in *I. hospitalis* [6]. An adjacent gene to thermosome subunit Iho0096 encodes an abundant small protein (Iho0095; 108 aa) that belongs to a ferritin-like domain family that includes a rubrerythrin domain. Rubrerythrin is an abundant protein present in other thermophiles that is also part of the constitutive oxidative stress response mechanism. Specifically, it has been shown to protect against peroxides in *Pyrococcus* [17]. Another abundant protein that could be categorized similarly is the universal stress family protein UspA (Iho0144), which, interestingly, was down-regulated in the co-culture with *N. equitans*. The gene is part of an operon that encodes two hypothetical proteins (Iho0142 and 0143) with no known homologs, which were also expressed at similar levels to UspA. Two FAD-dependent pyridine nucleotide-disulphide oxidoreductases (Iho0673 and 0899) were expressed at high levels as well. One of the two (Iho0673) was significantly up-regulated in the co-culture (∼5-fold). This family of enzymes may also be involved in protection against oxidative stress and regeneration of oxidized pyridine nucleotides [18]. Iho0673 belongs to a predicted three-gene operon that includes a conserved unknown protein (Iho0672) that is up-regulated ten-fold in co-culture and a sulfur oxidoreductase that is present at relatively unchanged levels. The significance of these genes' up-regulation in the presence of *N. equitans* is unclear but may indicate an increased level of cellular stress due to the association. Another protein potentially involved in cellular stress, an AAA-ATPase (Iho1431) is also present at high levels and up-regulated (2-fold) in co-culture as well. The other oxidoreductase, Iho0899 has a signal sequence, suggesting secretion into the periplasm and is one of the previously identified candidates of horizontal gene transfer with *N. equitans* [4]. Based on RSpC, it appears to be the most abundant *Ignicoccus* protein measured in the co-culture with *N. equitans* (RSpC 2387). Its close homolog in *N. equitans*, Neq024 is also a highly abundant protein. While the presence of high levels of stress response proteins and their apparent up-regulation in co-culture may indicate that *I. hospitalis* reacts negatively to the presence of *N. equitans*, further studies in different culture stages will be necessary to test this.

**Table 1:** The most abundant *I. hospitalis* proteins, based on the sum of RSpC values of three independent measurements on the pure culture (Igni) and the co-culture (Igni_Nano) samples as well as the ratio between the abundance in the pure culture versus the co-culture (I/I_N).

Gene	Protein name	arCOG	class	arCOG annotation	Size (aa)	Igni	Igni_Nano	I/I_N
Iho_0230	LSU ribosomal protein L7A	1751	J	Ribosomal protein HS6-type (S12/L30/L7a)	128	4106	2119	1.9
Iho_0899	FAD-dependent pyridine nucleotide-disulphide oxred	1065	R	NAD(FAD)-dependent dehydrogenase	367	2693	2387	1.1
Iho_1150	translation elongation factor 1A (EF-1A/EF-Tu)	1561	J	Translation elongation factor EF-1alpha (GTPase)	442	2178	2074	1.0
Iho_0560	thiazole-adenylate synthase	574	G	Ribulose 1,5-bisphosphate synth.	265	2114	1995	1.1
Iho_0174	nucleoid protein Alba	1753	K	Archaeal DNA-binding protein	97	1983	1147	1.7
Iho_1081	Archaeal ATP synthase subunit F	4102	C	Archaeal/vacuolar-type H+-ATPase subunit F	102	1836	774	2.4
Iho_0677	SSU ribosomal protein S6E	1946	J	Ribosomal protein S6E (S10)	236	1418	841	1.7
Iho_0363	D-fructose 1,6-bisphosphatase	4180	G	Archaeal fructose 1,6-bisphosphatase	387	1357	2145	0.6
Iho_0897	thermosome subunit	1257	O	Chaperonin GroEL (HSP60 fam.)	541	1301	1250	1.0
Iho_0096	thermosome subunit	1257	O	Chaperonin GroEL (HSP60 fam.)	558	1256	865	1.5
Iho_0459	1-Cys peroxiredoxin	312	O	Peroxiredoxin	234	1229	1965	0.6
Iho_0144	UspA domain protein	2053	T	Nucleotide-binding protein UspA	147	1208	581	2.1
Iho_0880	Inorganic diphosphatase	1711	C	Inorganic pyrophosphatase	187	1171	874	1.3
Iho_0143	protein with unknown function	-	-	-	66	1129	758	1.5
Iho_0595	Vinylacetyl-CoA Δ-isomerase	2143	Q	Aromatic ring hydroxylase	512	1097	1008	1.1
Iho_1363	heat shock protein Hsp20	1833	O	Molecular chaperone (HSP20	196	1092	476	2.3
Iho_0189	Ferredoxin-like protein	349	C	Ferredoxin	79	1078	500	2.2
Iho_0095	protein with unknown function	5908	S	Ferritin-like domain	108	1050	656	1.6
Iho_0936	6,7-dimethyl-8-ribityllumazine synthase	1323	H	Riboflavin synthase beta-chain	139	1045	1152	0.9
Iho_0274	phosphoglycerate kinase	496	G	3-phosphoglycerate kinase	408	1029	1286	0.8
Iho_0679	Archaeal ATP synthase subunit B	865	C	Archaeal/vacuolar-type H+-ATPase subunit B	471	981	1557	0.6
Iho_1305	Archaeal ATP synthase subunit A	868	C	Archaeal/vacuolar-type H+-ATPase subunit A	596	957	1059	0.9
Iho_0226	transcriptional regulator, AsnC	1117	K	Lrp/AsnC family C-term. domain	77	929	951	1.0
Iho_1359	Multiheme cytochrome	7617	C	Multiheme cytochrome	638	906	397	2.3
Iho_1243	V4R domain-containing protein	-	-	-	182	868	435	2.0
Iho_0142	protein with unknown function	-	-	-	171	858	1042	0.8
Iho_1367	nickel-dependent hydrogenase, large subunit	1550	C	Ni,Fe-hydrogenase I large subunit	664	847	1447	0.6
Iho_1431	AAA family ATPase, CDC48	1308	O	ATPase of the AAA+ class	729	781	1814	0.4
Iho_1266	Outer membrane pore protein	-	-	-	85	776	519	1.5
Iho_1383	translation elongation factor 2 (EF-2/EF-G)	1559	J	Translation elongation factor G, EF-G (GTPase)	740	770	934	0.8
Iho_1254	beta-lactamase domain protein	497	R	Zn-dependent hydrolase of the beta-lactamase fold	233	689	848	0.8
Iho_1113	phosphoenolpyruvate synthase	1111	G	Phosphoenolpyruvate synthase	821	653	1220	0.5
Iho_0748	Cobalamin-independent synthase MetE	1877	E	Methionine synthase II (cobalamin-independent)	332	650	1409	0.5
Iho_0256	AMP-dependent synthetase and ligase	4201	I	Acyl-coenzyme A synthetase/AMP-(fatty) acid ligase	412	521	836	0.6
Iho_1366	Nickel-dependent hydrogenase small subunit	2474	C	Ni,Fe-hydrogenase I small subunit	422	414	884	0.5
Iho_0673	FAD-dependent pyridine nucleotide-disulphide oxred	1064	R	NAD(FAD)-dependent dehydrogenase	385	314	1618	0.2
Iho_0929	Roadblock/LC7 family protein	2603	R	Roadblock/LC7 domain	123	261	833	0.3

Proteins and molecular complexes responsible for energy generation were also detected at significant levels. All the archaeal ATP synthase subunits were identified, except for the transmembrane subunit c (Iho0682), which forms the oligomeric ring. That protein is relatively small (113 aa) and upon sequence analysis we determined its predicted tryptic peptides have sizes outside the detection criteria, perhaps explaining its absence. The A and B subunits (Iho1305 and 0679), on the other hand, were found at high, equivalent levels (average RSpC 957 and 981), reflecting their equimolar multimeric involvement in the ATP synthase architecture. The other subunits were also detected at moderate to high abundance except for subunit a (Iho0609), which is an integral membrane protein and may have been inefficiently recovered during initial protein extraction. Subunit F (Iho1081), while very abundant (average RSpC 1836), appears down regulated two-fold in the presence of *N. equitans*. The *I. hospitalis* ATP synthase has recently been shown to be located on the outer cell membrane [19]. Another energy-related membrane complex detected at high levels, Ni-hydrogenase, was up-regulated two-fold in the presence of *N. equitans*. The four proteins predicted to form the complex (small and large hydrogenase subunits, a 4Fe-4S ferredoxin, and a transmembrane membrane protein) are encoded by genes that appear to be part of a single transcriptional unit (Iho1366–1369); all constituents were detected though the transmembrane protein was identified at very low levels, probably due the aforementioned tryptic incompatibility. Unlike ATP synthase and Ni-hydrogenase, the sulfur reductase appears to be less abundant. This complex has been previously shown by antibody staining to be located on the outer membrane like ATP synthase [19]. Though its exact architecture remains to be characterized, there appears to be two operons encoding potential protein subunits, Iho0801–0803 and Iho0528–0530, the latter possibly of bacterial origin [4]. In the *I. hospitalis* sample, the proteins encoded by Iho0801–0803 were present at low to moderate levels (RSpC 2–60) while their abundance was even lower in the co-culture, suggesting a more than five-fold down-regulation by the presence of *N. equitans*. The proteins encoded by Iho0528–0530 were slightly more abundant (RSpC 13–174) and their level increased approximately two-fold in the presence of *N. equitans*. Another membrane complex that so far has not been associated with any cellular function is the outer membrane pore, assembled by the oligomerization of Iho1266 [20]. This abundant protein constituent of the cell [6] appears to be slightly down-regulated (1.5 fold) in co-culture. While its involvement in the transfer of metabolites between *I. hospitalis* and *N. equitans* is uncertain, clearly the presence of *N. equitans* does not induce a significant increase in the number of pores on the host outer membrane.

Among the proteins involved in genetic information processing, the large subunit of ribosomal protein L7AE (Iho0230) was the most abundant protein identified in the *I. hospitalis* pure culture (RSpC 4160) but was down regulated two-fold in the presence of *N. equitans*. L7AE is an important, multifunctional RNA-binding protein that recognizes the K-turn motif in ribosomal box H/ACA and box C/D sRNAs, and is thus involved in RNA modification pathways. As will be discussed further, other proteins involved in RNA synthesis and control are down-regulated as well in the co-culture. Additional proteins of high abundance involved in translation and transcription include the elongation factors 1A and 2 (Iho1150 and Iho1383) as well as an AsnC family transcription factor (Iho0226), all of which are present at similar levels in both pure and co-culture. Among the abundant proteins involved in chromosomal structure and cell division are the nucleoid protein Alba (Iho0174) and a putative membrane associated protein involved in DNA segregation, annotated as a multiheme protein (Iho1359); both proteins are down-regulated in the co-culture. A protein that is up-regulated three-fold in the presence of *N. equitans* is Iho0929, a member of the roadblock/LC7 superfamily. The function of these proteins in prokaryotes is not known but it has been suggested to be involved in regulation of GTPase activity in the cell [21].

Abundant metabolic enzymes include acetate-CoA ligase (Iho0256, RSpC 574), which likely provide an additional source of acetyl-CoA using acetate. In the original genome, two adjacent genes (Iho0256 and 0257) encoded separate regions of the enzyme. We have re-sequenced that region of the genome and determined that in fact a sequencing error was responsible for the initial split of that enzyme in two separate ORFs. The genomic sequence deposited in GenBank and the gene annotation is being corrected. A recent independent analysis of that gene also reached the same conclusion and also conformed that Iho0256 can use acetate as a substrate [22]. Other abundant enzymes important to central metabolism were identified including phosphoglycerate kinase (Iho0274), fructose 1,6-bisphosphatase (Iho0363), 4-hydroxybutyryl-CoA dehydratase (Iho0595), enzymes involved in the biosynthesis of thiamin and riboflavin (Iho0560 and Iho936) as well as in amino acid metabolism (the two subunits of methionine synthase, Iho0747 and 0748). Phosphoenolpyruvate synthase (Iho1113), a key enzyme in carbon fixation, is up-regulated two fold in co-culture, potentially reflecting the increased metabolic demand imposed by the association with *N. equitans*. Similarly, the pyruvate ferredoxin oxidoreductase complex, which fixes CO_2_ to pyruvate and is encoded by a four-gene operon (Iho1256–1259), is also up-regulated close to two-fold in co-culture. Phosphoenolpyruvate carboxylase (Iho0341) also follows this trend. Though perhaps indicative of increased metabolic and energetic demand put upon *I. hospitalis* by its association with *N. equitans*, it remains to be seen whether modest two-fold variations in the amount of individual proteins or complexes involved in critical steps of carbon fixation and energy generation are biologically significant for the relationship between the two organisms.

### The *N. equitans* proteome

As described above, differential proteomics based on the proteome derived from co-culture purified *N. equitans* may not be as biologically informative as compared to *I. hospitalis*. Nevertheless, the measured proteome provides a glimpse into the metabolic processes undertaken by this obligate ectoparasite. Structural proteins, regulators and enzymes involved in packaging and processing of the genetic material were found in high abundance both in the purified *N. equitans* sample and in the co-culture. These include the archaeal histone Neq348 (for which in the purified *N. equitans*, we observed the highest number of rebalanced normalized spectral counts, RSpC 9540) and the nucleoid protein Alba (Neq363), components of the replication, recombination and repair machineries (Neq537, 426, and possibly 368), as well as the cell division proteins FtsZ (Neq133) and MinD (Neq119). Several ribosomal proteins, translation elongation factor 1A, as well as two transcription factors (Neq098 and 534) were also among the top 30 most abundant proteins (**Table 2**).

**Table 2:** The most abundant *N. equitans* proteins, based on the sum of RSpC values of the three independent measurements for the *I.hospitalis*-*N. equitans* co-culture (Igni_Nano) and the purified *N. equitans* sample (Nano).

Gene	Protein Name	arCOG	Class	Archaeal COG annotation	Size (aa)	Igni_Nano	Nano
NEQ348	archaeal histone	2144	L	Histones H3 and H4	75	18292	9540
NEQ300	S-layer protein				941	7948	7265
NEQ174	KaiC domain protein	1171	T	RecA-superfamily ATPase implicated in signal transduction	256	3478	3048
NEQ288	archaeal histone	2144	L	Histones H3 and H4	82	2245	2500
NEQ319	LSU ribosomal protein L7AE	1751	J	Ribosomal protein HS6-type (S12/L30/L7a)	125	1232	2337
NEQ026	protein with unknown function	6945	S	Uncharacterized conserved protein	102	2789	2303
NEQ082	translation elongation factor 1A (EF-1A/EF-Tu)	1561	J	Translation elongation factor EF-1alpha (GTPase)	433	2850	2260
NEQ461	Inorganic pyrophosphatase	1711	C	Inorganic pyrophosphatase	160	1827	2072
NEQ368	Putative endonuclease	6583	L	Endonuclease IV	293	1390	2066
NEQ133	cell division protein FtsZ	2201	D	Cell division GTPase	355	1668	1848
NEQ363	nucleoid protein Alba	1753	K	Archaeal DNA-binding protein	90	1831	1757
NEQ426	RecA/RadA recombinase	415	L	RecA/RadA recombinase	325	1008	1352
NEQ141	thermosome subunit	1257	O	Chaperonin GroEL (HSP60 family)	540	2187	1327
NEQ537	archaeal DNA polymerase sliding clamp	488	L	DNA polymerase sliding clamp subunit (PCNA homolog)	254	1472	1299
NEQ344	heat shock protein Hsp20	1832	O	Molecular chaperone (HSP20 family)	136	1031	1277
NEQ098	Predicted transcriptional regulator	2037	K	Sugar-specific transcriptional regulator TrmB	276	927	1254
NEQ236	S-layer-associated protein				336	1263	1171
NEQ221	protein with unknown function				98	1186	1116
NEQ449	protein with unknown function				164	1567	1082
NEQ534	Predicted transcriptional regulator	921	K	Predicted transcriptional regulator	147	2395	1071
NEQ386	Dinitrogenase iron-molybdenum cofactor-domain	2737	S	NifX family protein	118	729	1019
NEQ207	LSU ribosomal protein L13P	4242	J	Ribosomal protein L13	162	638	986
NEQ258	protein with unknown function	2155	R	Protein implicated in RNA metabolism, PRC-barrel domain	85	976	984
NEQ277	protein with unknown function	2263	D	Predicted cell division protein, SepF homolog	125	887	984
NEQ024	protein with unknown function	1065	R	NAD(FAD)-dependent dehydrogenase	365	471	876
NEQ446	SSU ribosomal protein S9P	4243	J	Ribosomal protein S9	136	816	863
NEQ119	septum site-determining protein MinD	589	N	Antiactivator of flagellar biosynthesis FleN, an ATPase	244	569	845
NEQ535	aspartyl-tRNA synthetase	406	J	Aspartyl/asparaginyl-tRNA synth.	404	512	842
NEQ190	branched chain aa aminotransferase apoenzyme	2297	E	Branched-chain aa aminotransferase	298	642	835
NEQ203	proteasome endopeptidase complex, beta domaint	970	O	20S proteasome, beta subunit	196	232	829

Among the most abundant enzymes identified is an inorganic phosphatase (Neq461; RSpC∼2000), likely important for preventing a buildup of intracellular pyrophosphate resulting from nucleic acid biosynthesis, aminoacyl-tRNA synthetases (e.g. Neq535), glutamate dehydrogenase (Neq077) and nucleoside diphosphate kinase (Neq307), which can regenerate the NTP pool using ATP. These proteins appear to enable important metabolic transformations that cannot be provided by the host and led to the maintenance of endogenous *N. equitans* genes even though the majority of other biosynthetic functions were lost.

Protein processing and turnover are highly active as well, as evidenced by the abundance of chaperones (Neq141 and 344), the proteasome (Neq203), and several other peptidases. On the cell surface, the S-layer protein (Neq300) and its associated, smaller companion Neq236 dominate (RSpC>7000 and >1000, respectively). Several of these proteins have previously been identified in isolated membrane fractions that contained contact point between the two organisms [7].

Though the above mentioned abundant proteins are physiologically important to *N. equitans*, it is more likely that proteins involved in host recognition, cell surface interaction, transfer of metabolites, and/or other types of membrane proteins would provide more pertinent biological insights with regard to the association. As follows, many of the expressed *N. equitans* proteins, including predicted membrane proteins, have no recognizable homologs. Several of those identified at high levels include Neq035, 099, 222 and 492. Proteins that cannot be assigned a clear function in *N. equitans* but have been classified to existing microbial families include those involved in signaling and potential cell-cell interaction. Several members were detected at high or moderate levels, including a RecA-superfamily ATPase, implicated in signal transduction and containing the KaiC domain (Neq174, RSpC>3000), and two Flp pilus proteins (Neq267 and 268). Identifying the functions of all these proteins and uncovering the mechanism of interaction with the *Ignicoccus* host will clearly require more in-depth biochemical and ultrastructural inquiry.

### *I. hospitalis* proteome changes induced by *N. equitans*

Among the major questions surrounding the *I. hospitalis-N. equitans* relationship is how the presence of *N. equitans* impacts its host's physiology. By identifying changes in *I. hospitalis* protein expression occurring when *N. equitans* is present on its surface, relative to its solitary growth, mechanisms that enable this association should become apparent. In fact, defining the very nature of the association requires such observations. Here we provide a first view of the proteomic changes that occur in *I. hospitalis* as *N. equitans* populates its surface.

To evaluate the impact that *N. equitans* has on its host proteome at broad cellular activities level we have employed a Gene Set Enrichment Analysis (GSEA) [23], [24] to measure the variation of *I. hospitalis* arCOG categories between the two culture conditions (presence or absence of *N. equitans*). **Figure S2** summarizes the result of the GSEA, where positive normalized enrichment scores (NES) reflect the degree to which an arCOG category is over-represented (enriched) in the *I. hospitalis-N. equitans* co-culture while negative scores reflect over-represented categories in the pure culture relative to the co-culture. Overall, in the co-culture, GSEA points especially to increased cell cycle control, energy generation, post translation protein modification/turnover and membrane biogenesis accompanied by a decrease in transcription and replication functions. These broad responses are perhaps expected in the culture stage where *N. equitans* numbers are high and the host has largely ceased cell division and has to cope with its companion's demand. Further individual analyses of the most abundant *Ignicoccus* proteins were performed in order to dissect its response to *Nanoarchaeum* in greater detail.

**Figure 3:**
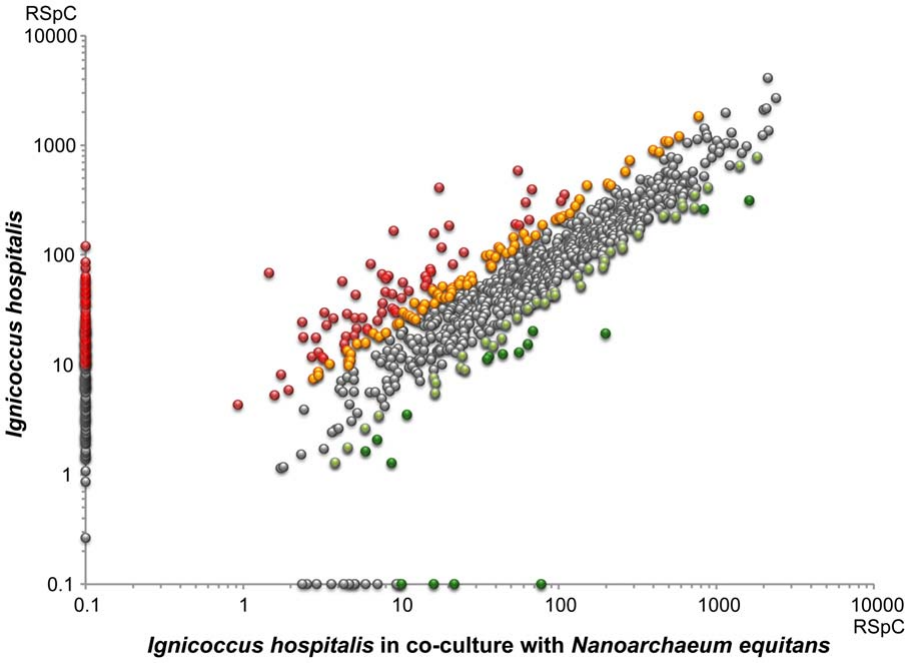
Changes in *I. hospitalis* relative protein abundance between the pure culture and the co-culture with *N. equitans*. Proteins up-regulated in the co-culture are in dark green (>3 fold) or light green (2–3 fold). Down regulated proteins are in red (>3 fold) or orange (2–3 fold). Proteins that were only detected in one of the sample types are represented on either axis and an arbitrary spectral count (RSpC) threshold >10 was chosen for coloring. The scatter plot uses the sum of spectral counts for each protein between the three independent measurements for each sample.

Among the detected *I. hospitalis* proteins, the relative abundance of approximately 10% (106 proteins) differed by 2-fold or more between the pure and co-culture (**Figures 3** **,** **4** and **Tables 3** **,** **4**; see **Table S1** for the relative fold-change values for all detected proteins). As presented above, several major players involved in energy generation, including the constituents of *I. hospitalis'* ATP synthase, Ni-Fe hydrogenase, and polysulfide reductase appear to be up regulated upon *N. equitans'* association. The assumed increased rate of respiration and ATP synthesis in *I. hospitalis* suggests an environment with increased energy demands, most likely resulting from the metabolic needs of *N. equitans* which itself does not provide compensation for, at least energetically speaking. Increased levels of key central metabolic enzymes of *I. hospitalis* support this postulate as well. Several of these enzymes provide key substrates for multiple biosynthetic pathways as well as for carbon fixation, including acetyl-CoA synthase (Iho256), pyruvate ferredoxin-oxydoreductase complex (Iho1256–1259), phosphoenolpyruvate synthase (Iho113), phosphoenolpyruvate carboxylase (Iho341), and fructose 1,6-bisphosphatase (Iho363). Their up-regulation in the co-culture may indicate an increased respiratory and basal metabolic burden brought forth by multiple *N. equitans* populating each *I. hospitalis* cell. All these inferences will certainly require biochemical validation and additional quantitative proteomic measurements in various co-culture stages.

**Figure 4:**
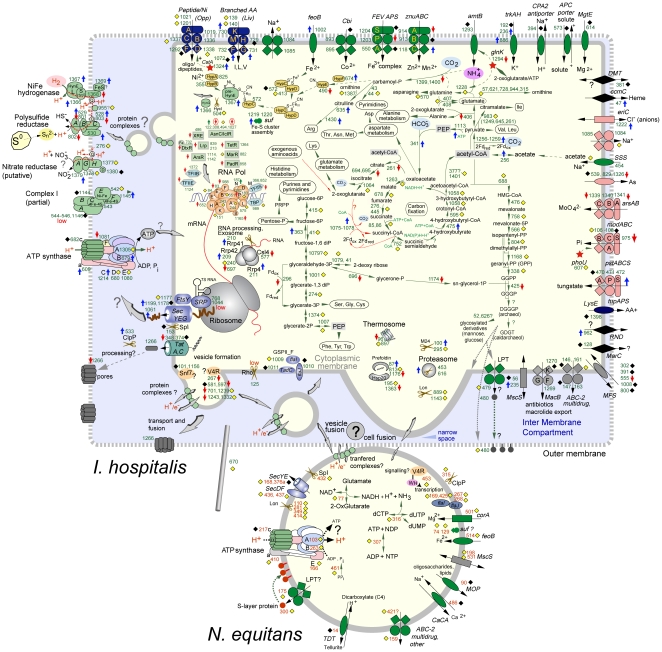
Updated reconstruction of *I. hospitalis-N. equitans* metabolism and interaction (modified from [**4**]), incorporating results of recent physiological and ultrastructural studies [**15]**, [20****]. The localization of most *I. hospitalis* membrane complexes (outer vs. inner membrane) is unknown and arbitrarily depicted here as spanning both membranes. *I. hospitalis* proteins detected by proteomics are indicated by yellow diamonds (<1.5 fold change between pure culture and co-culture) or blue or red arrows (>1.5 fold increase or decrease, respectively in co-culture versus single culture). Black diamonds indicate proteins that were not detected. In *N. equitans*, the variation between the purified sample and the co-culture values are not being represented, the yellow/black diamonds only indicate detection/non-detection.

**Table 3:** *I. hospitalis* proteins up-regulated by the presence of *N. equitans*.

Gene	Product Name	SigP	PredS	TMD	Igni_Nano	Igni	I_N/I
Iho_1061	Sec61beta				77	0	>50
Iho_0672	protein of unknown function DUF1641				198	19	10
Iho_0673	FAD-dependent pyridine nucleotide-disulphide oxidoreductase				1618	314	5
Iho_0528	polysulphide reductase, NrfD			Yes	56	13	4
Iho_0472	ABC-type tungstate transport system permease component-like protein	Yes	Yes	Yes	64	15	4
Iho_0356	protein with unknown function				69	20	3
Iho_0929	Roadblock/LC7 family protein				833	261	3
Iho_0588	SSU ribosomal protein S19P				727	269	3
Iho_1052	protein with unknown function	Yes	Yes	Yes	138	52	3
Iho_0087	prefoldin, alpha subunit				191	77	2
Iho_0704	phosphoribosyltransferase				554	224	2
Iho_1431	AAA family ATPase, CDC48 subfamily	Yes			1814	781	2
Iho_0906	glutaredoxin-like domain protein				196	87	2
Iho_1258	pyruvate ferredoxin oxidoreductase, alpha subunit	Yes			613	275	2
Iho_0235	protein with unknown function			Yes	82	36	2
Iho_1259	pyruvate ferredoxin oxidoreductase, beta subunit				255	115	2
Iho_0257	acyl-coenzyme A synthetase/AMP-(fatty) acid ligase-like protein				95	43	2
Iho_0748	cobalamin-independent synthase MetE				1409	650	2
Iho_1366	Nickel-dependent hydrogenase small subunit	Yes	Yes		884	414	2
Iho_0389	protein with unknown function				194	91	2
Iho_1077	2-oxoglutarate ferredoxin oxidoreductase, beta subunit				320	153	2
Iho_1145	4Fe-4S ferredoxin, iron-sulfur binding domain protein	Yes			155	74	2
Iho_0066	FAD-dependent pyridine nucleotide-disulphide oxidoreductase	Yes			132	63	2
Iho_0747	methionine synthase (B12-independent)				729	352	2
Iho_0148	protein with unknown function				252	123	2
Iho_0609	H(+)-transporting two-sector ATPase			Yes	94	46	2
Iho_0533	protein of unknown function DUF114				461	226	2
Iho_0892	prephenate dehydrogenase/chorismate mutase				89	43	2
Iho_1369	4Fe-4S ferredoxin, iron-sulfur binding domain protein				474	234	2
Iho_0984	protein with unknown function				603	299	2
Iho_1324	carbon starvation protein CstA			Yes	54	27	2

**Table 4:** *I. hospitalis* proteins down-regulated by the presence of *N. equitans*.

Gene	Product Name	SigP	PredS	TMD	Igni-Nano	Igni	I_N/I
Iho_0514	protein with unknown function	Yes	Yes		152	430	0.35
Iho_0234	protein with unknown function				103	310	0.33
Iho_0974	Cupin 2, conserved barrel domain protein				65	208	0.31
Iho_0406	aminotransferase, class I and II				109	355	0.31
Iho_1248	3-isopropylmalate dehydratase, small subunit				56	186	0.30
Iho_1221	protein with unknown function				53	190	0.28
Iho_0260	molybdopterin synthase subunit MoaD/E				14	52	0.27
Iho_0490	hydrogenase expression/formation protein HypE				21	82	0.26
Iho_0794	ribosomal biogenesis protein, containing Brix domain				15	59	0.25
Iho_0366	DNA-directed RNA polymerase, subunit M				25	105	0.24
Iho_0170	protein with unknown function				14	64	0.22
Iho_1049	protein with unknown function				16	70	0.22
Iho_0949	Predicted metal-sulfur cluster biosynthetic enzyme				62	301	0.21
Iho_0930	protein with unknown function				10	56	0.18
Iho_0802	molybdopterin oxidoreductase				8	46	0.17
Iho_1027	transcriptional regulator, AsnC family				68	397	0.17
Iho_0978	Small nuclear ribonucleoprotein, LSM family				18	117	0.16
Iho_0801	4Fe-4S ferredoxin, iron-sulfur binding domain protein				8	60	0.13
Iho_0780	protein with unknown function				8	64	0.13
Iho_0169	protein with unknown function				8	66	0.11
Iho_1032	aldehyde ferredoxin oxidoreductase				20	185	0.11
Iho_1302	protein with unknown function				16	157	0.10
Iho_0975	phosphate ABC transporter substrate-binding protein	Yes	Yes	Yes	55	587	0.09
Iho_0355	hydrogenase accessory protein HypB				6	83	0.08
Iho_0280	3-dehydroquinate dehydratase				4	57	0.07
Iho_0054	protein with unknown function				9	166	0.05
Iho_0006	protein with unknown function				17	410	0.04
Iho_0025	aldehyde ferredoxin oxidoreductase				1	69	0.02
Iho_0858	transcriptional regulator, PadR family				<1	36	0,02
Iho_1223	protein with unknown function				<1	54	0.02
Iho_1165	DNA-directed RNA polymerase, subunit RPB8				<1	55	0.02
Iho_0175	protein with unknown function				<1	58	0.02
Iho_1088	protein with unknown function				<1	61	0.02
Iho_1289	protein with unknown function				<1	63	0.02
Iho_0083	DNA-directed RNA polymerase, subunit E″				<1	76	0.01
Iho_0458	anaerobic rNTP reductase activating protein				<1	85	0.01
Iho_0308	transcriptional regulator, AsnC family				<1	120	0.01

Among the genetic information processing steps, transcription was negatively impacted the most in the co-culture sample. Multiple subunits of the RNA polymerase complex were significantly reduced (RpoE″, F, H, Rpb8), as were important general transcriptional regulators, such as the initiation (TFIIB, E) and elongation factors (TFIIS). Almost all transcription factors were decreased, some >5 fold (e.g. Iho1027, 0858), with the most impacted being an AsnC family regulator (Iho0308) which was reduced from an RSpC of 120 in the pure *I. hospitalis* culture to below detection in the co-culture. As follows, perhaps only one transcription factor may be specific for the co-culture state, Iho0122 (AsnC family), although detected at a low level. Gene expression and promoter activity analyses will be required in order to determine the significance of these changes in the transcriptional machinery, specifically in how they affect global transcripts levels and/or correlate to measured protein abundance.

Aside from the variations in transcription factor abundance, several other global cellular regulators are significantly different in the *I. hospitalis-N. equitans* co-culture. These differences include a two-fold increase in the carbon starvation protein (CstA, Iho1324) and a three-fold increase in a relatively abundant protein, the roadblock/LC7 superfamily protein Iho0929. The function of RLC7 proteins in Bacteria and Archaea is unclear but has been proposed to involve regulation of cellular GTPases [21]. Another important global metabolic regulator, the P_II_ signal transduction protein encoded by *glnK* (Iho1294), was present at two-fold lower level in the co-culture. GlnK is widely present in Bacteria and Archaea and has complex regulatory functions that integrate energy, carbon and nitrogen metabolism [25]. Binding of 2-oxoglutarate and ATP can induce conformational changes that impact the interaction of GlnK with a variety of cellular targets (e.g. enzymes involved in nitrogen metabolism, the ammonium transporter, and transcription factors). By sensing the levels of 2-oxoglutarate and glutamate, both substrates for ammonia assimilation, GlnK can induce or repress genes involved in the global nitrogen cycle. Details of this regulatory control in *Ignicoccus* and the significance of GlnK down-regulation in the presence of *N. equitans* are still unknown. Potentially linked to that was an observed reduced abundance of several enzymes involved in nitrogen and amino acid/oxoacids metabolism was observed, including carbamoyl-phosphate synthase (Iho1399, 1400), an aminotransferase (Iho0496) and two aldehyde ferredoxin oxidoreductases (Iho25, 1032).

As already discussed, the stress response and protein processing machinery of *I. hospitalis* has a mixed response to the presence of *N. equitans*, at least with regard to the samples characterized in this study. Most protein chaperones are either unchanged or decreased in co-culture (e.g. hsp20 [Iho1363] and a prefoldin [Iho1176]), perhaps indicating a reduced rate of protein synthesis. Interestingly, the translation initiation factor Iho0676 is reduced as well. However, one prefoldin (Iho0087) is elevated ∼2.5 fold, suggesting a potential functional differentiation among the related chaperones. The proteasome (Iho0453, 0616) and a few proteases (Iho0100, 0533) were also modestly elevated (<2-fold) in the co-culture. Most notable, however, was the >5-fold increase in the abundant protein FAD-dependent pyridine nucleotide-disulphide oxidoreductase (Iho0673) and its neighbor (Iho0672), a protein with unknown function. To understand the specificity and the role of these proteins in *I. hospitalis* association with *N. equitans* it will be important to determine whether the dynamic response of this pair of gene products is integral to the establishment and/or progression of the co-culture.

The mechanisms that enable the transfer of metabolic products (e.g. amino acids, lipids, nucleotides) from *I. hospitalis* to *N. equitans* are completely unknown at this time. Even though the sample analyzed here contained *N. equitans* in stationary phase, perhaps implying diminished growth and thus metabolic uptake, the proteomic datasets were scrutinized to uncover potential clues to the mechanisms of interaction and/or communication between the two organisms. In particular, we analyzed proteins potentially involved in signaling, defense and transport. Based on homology, the *I. hospitalis* genome encodes relatively few proteins that can be assigned to these functional categories. Such proteins may be constitutively present or be induced in co-culture as a result of signaling/sensing events. Among the cellular defense mechanisms, the *I. hospitalis* genome contains nine CRISPR repeat sequences and 37 predicted CAS genes, some of them grouped in distinct operons (Iho0326–0329, Iho0460–0464 and Iho1139–1141). Most of these proteins were detected with several at moderate to high levels (RSpC>100). However, they appear to not be differentially expressed, suggesting the CRISPR defense mechanism is persistently active in this organism or at least does not respond to *N. equitans* populating its surface. Since the CRISPR system acts in protecting against both phage infection and presence of foreign DNA (a process that is not expected to occur upon *N. equitans* attachment), the absence of a CRISPR response is not surprising. Another noteworthy protein that responded to the presence of *N. equitans* is Sec61 (Iho1061), a component of the protein secretion apparatus. This protein was only detected (RSpC of 77) in the co-culture and, even though there is yet no evidence of protein transfer from *Ignicoccus* to *Nanoarchaeum*, the general protein secretion apparatus may be employed during metabolite transfer. Several other membrane proteins with unknown function were also either enriched (Iho1052, 0235) or depleted (Iho0556, 0514) in the co-culture.

### Correlations between the proteomic data and genomic operon structure

In Bacteria and Archaea, most genes are organized in co-transcribed and co-regulated clusters of genes or operons. Most operons are not conserved over long evolutionary distances as they are generally under the pressure of genome rearrangement and fragmentation by insertion of mobile elements [26]. In many cases, genes that belong to the same operon encode separate interacting subunits of enzymatic complexes or perform coupled metabolic transformations. Even though post-transcriptional and post-translational regulation could significantly impact the synthesis and steady-state level of the individual proteins encoded by the genes of an operon, intuitively the relative abundance of such proteins would be expected to correlate with the operon structure. The application of multiple complementary approaches to dissect the functional complexity of microbial genomes indicates that additional regulatory processes, including multiple transcription start sites within an operon, antisense and regulatory RNAs, can complicate *in silico* annotation and functional prediction [27]. Proteomic data has already been successfully used to verify and predict both operons and regulons in some Archaea [9] as well as to pinpoint the existence of significant post-transcriptional regulatory mechanisms that impact the protein abundance of co-transcribed genes [28], [29].

**Figure 5:**
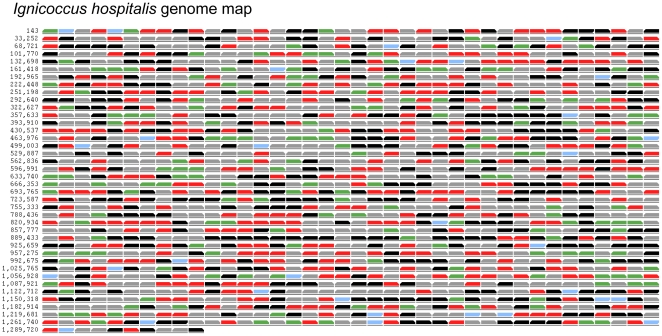
The *I. hospitalis* proteome and the effect of co-culture with *N. equitans* at the genome level. Genes are arranged in rows with the coordinates on the left corresponding to the nucleotide position in the genome. Blue indicate RNA genes. In black are genes encoding proteins that were not detected. Green and red correspond to proteins that were up- or down-regulated, respectively, by 1.5 fold or more between the co-culture versus the independent culture samples. Grey indicates proteins with <1.5 fold variation between the samples. The direction of the slanted rectangle indicates the direction of transcription. Not all consecutive genes transcribed in the same direction are predicted to be in the same transcriptional unit (operon).

One of the genomic characteristics shared by both *N. equitans* and *I. hospitalis* is the absence and/or fragmentation of many relatively long and conserved operons that are present in other Archaea and Bacteria, e.g. those that contain ribosomal protein genes. Nevertheless, there are still a sufficient number of predicted, relatively conserved operons, which allows for matching the average abundance of predicted proteins to an operon-specific response brought about by the association of these two Archaea. Considering each operon as a gene set, we used the GSEA [24] to find operons differentially expressed between the two culturing conditions, *I. hospitalis* grown by itself or in co-culture with *Nanoarchaeum*. Between those two biological conditions, only a limited number of individual proteins changed by a factor greater than two–fold. Thus, it came as no surprise that only few operons responded significantly to the association. Such operons included those that contain genes strongly up- or down-regulated by the presence of *N. equitans* (Iho0672–0673, Iho0169–0170, Iho1087–1088), with several encoding interacting subunits of the same enzymatic complex (i.e. NiFe hydrogenase and sulfur reductase). An important result also included operons and/or clusters of transcriptionally independent but adjacent genes that appeared silent, i.e. their encoded proteins were not detected (**Figure 5**). Among them is a predicted daunorubicin resistance ABC transporter (Iho0146–0147), an operon containing predicted type II secretion system proteins (Iho1009–1016), and an operon containing five CRISPR-associated protein genes (Iho1132–1135). These sporadically occurring examples could indicate the presence of regulatory mechanisms that prevented expression of these genes under the culture conditions utilized in this study, perhaps precluding their proteomic observation unless exposed to specific environmental challenges. Notable however were multiple operons consisting of clusters of unexpressed genes that encoded hypothetical proteins with no recognizable function (e.g. Iho0262–0264, Iho0432–0437, Iho0806–0811, Iho0893–0896, Iho1023–1026, Iho1312–1316). Future experimental and comparative genomic analyses will be required to determine the evolutionary history and the function of these unrepresented operons and their encoded proteins.

### *I. hospitalis* proteins in *N. equitans*?

Initial genomic analysis pointed to a strict dependence of *N. equitans* on *I. hospitalis* for energetic and biosynthetic precursors (nucleotides, amino acids, lipids, sugars) [15]. Experimental evidence for the transfer of several types of molecules has indeed been obtained [2], [3], even though the mechanisms are still completely unknown. A major lingering question regards the transfer of large macromolecules (proteins, RNAs) that would enable *N. equitans* to perform functions not encoded by its own genome. At present, ultrastructural investigations of the *I. hospitalis-N. equitans* system have revealed features that could accomplish molecular transfer in two distinct ways [20], [30], [31]. Small vesicles that engulf cytoplasmic content and migrate through the large inter-membrane compartment (IMC) to the outer-membrane, independent of *N. equitans* presence, represents a relatively non-specific mechanism. This *I. hospitalis*-based cellular process of yet unknown physiological significance may have been exploited by *N. equitans* in its evolution as an ectosymbiont/parasite and could cargo not only small molecules but proteins as well, including membrane protein complexes. Alternatively, specialized structures (pores or channels) [20], [30] could form at the point of contact between the two organisms, which might facilitate the transfer and, depending on its size and functional mechanism, could control the types of molecules that pass from *I. hospitalis* to *N. equitans*. While a previous study focused on identifying protein complexes that form at the point of contact between the two organisms found a significant number of membrane and non-membrane associated proteins, the molecular specifics of the association remained unclear [7].

In the current study we attempted to address the question of whether or not *I. hospitalis* proteins may become integrated with the *N. equitans* cell. It has been shown that towards the end phase of the co-culture, a large fraction of *N. equitans* cells are released from the surface of *I. hospitalis* [2]. There is potential that a transfer of host proteins may enable them to survive for a limited time once detached from their host. This possibility remains speculative as it has not been determined if these free *N. equitans* cells are metabolically active or even able to re-attach to new host cells. Nevertheless, free *N. equitans* cells can be purified from their hosts. To perhaps provide evidence for *I. hospitalis* protein transfer, the proteome from a purified *N. equitans* cell fraction from a late co-culture stage was similarly measured. To assess the purity of the *N. equitans* cell fraction, the level of *I. hospitalis* cell carry-over was analyzed by both quantitative PCR and fluorescence in situ hybridization [1]; it was determined to be <1% *I. hospitals* relative to *N. equitans* (not shown).

Using the same criteria for peptide identification and assignment as described above, 464 *I. hospitalis* proteins were observed in at least one of the three independent MS runs performed on purified *N. equitans*. A total of almost 12,000 spectra were assigned to *I. hospitalis* proteins, as compared to 120,000 spectra assigned to *N. equitans* proteins. The relative abundance of most identified *I. hospitalis* proteins was very low, with only 170 proteins having a combined rebalanced normalized spectral count (RSpC) value of 20 or above, 22 of which having a RSpC of over 100 (**Table 5**). Two opposing possibilities may explain the existence of the observed *I. hospitalis* proteins. The first possibility is that the detected *I. hospitalis* proteins are contaminants brought through to the purified *N. equitans* cell sample and, thus have not been transferred specifically from the host. In the late culture phase, numerous *I. hospitalis* cells have already lysed resulting in their contents being released into the culture medium. Despite the purification steps, a low-level of the soluble host proteins could still remain trapped with the *N. equitans* cell mass, perhaps by non-specific binding to the cell surface, and be detected, though at low levels, by the highly sensitive MS approach. *I. hospitalis* membrane fragments could also co-purify with the *N. equitans* cells and, since the DNA and ribosomes would remain mostly in solution, the assays used for estimating co-purification of intact *I. hospitalis* cells may not fully reflect the efficiency of the purification procedure. At the same time, however, some of the free *N. equitans* cells may have became isolated by host cell lysis and not by self-detachment. In that case, they would carry host membrane fragments that may be stably and specifically interacting with *N. equitans* through the hypothetical interacting complex. Finally, the opposing possibility explaining the existence of *I. hospitalis* proteins suggests that some of the detected *I. hospitalis* proteins may be a result of specific intercellular transfer and represent integral constituents of *N. equitans*.

**Table 5:** *I. hospitalis* proteins enriched in the *N. equitans* sample.

Gene	Product Name	arCOG	class	SigP	PredS	TMD	Size (aa)	Igni_Nano	Nano	Ø
Iho_0801	4Fe-4S ferredoxin, iron-sulfur binding domain	437	C				219	5	46	55.7
Iho_0802	molybdopterin oxidoreductase (putative sulfur reductse subunit A)	243	C				834	5	24	28.7
Iho_0670	cell surface appendage protein			Yes	Yes	Yes	310	133	190	9.0
Iho_0731	amino acid/amide ABC transporter ATP-binding protein	411	E				254	57	76	8.4
Iho_0740	protein with unknown function						108	78	88	7.1
Iho_0066	FAD-dependent pyridine nucleotide-disulphide oxidoreductase	1252	C	Yes			357	86	90	6.6
Iho_0609	H(+)-transporting two-sector ATPase	1269	C			Yes	654	61	50	5.2
Iho_0249	SSU ribosomal protein S7P	49	J				195	93	68	4.6
Iho_1145	4Fe-4S ferredoxin, Fe-S binding dom.	1143	C	Yes			189	101	72	4.5
Iho_0988	protein with unknown function						117	71	51	4.5
Iho_0180	SSU ribosomal protein S13P	99	J				152	45	31	4.3
Iho_0914	ABC-type metal ion transporter periplasmic component/surface adhesin-like protein	803	P	Yes	Yes		277	31	21	4.3
Iho_1080	H+-transporting ATPase, E subunit	1390	C				208	76	48	4.0
Iho_1027	transcriptional regulator, AsnC family	1522	K				79	43	27	4.0
Iho_0680	H+-ATPase subunit D	1394	C				214	121	71	3.7
Iho_0529	sulfide reductase, subunit B	437	C				353	196	111	3.6
Iho_1369	4Fe-4S ferredoxin, iron-sulfur binding domain protein	247	C				479	308	172	3.5
Iho_0472	ABC-type tungstate transport system permease component-like protein	2998	H	Yes	Yes	Yes	287	41	23	3.5
Iho_1214	H+-ATPase subunit C	1527	C			Yes	341	198	101	3.2
Iho_0118	ABC transporter related	1121	P				247	53	26	3.2
Iho_1366	Nickel-dependent hydrogenase small subunit, N-terminal domain protein	1740	C	Yes	Yes		422	575	268	3.0
Iho_1378	putative nitrate reductase, subunit G	437	C	Yes			320	57	26	2.9
Iho_0732	amino acid/amide ABC transporter ATP-binding protein 2	410	E				246	112	48	2.7
Iho_0926	phosphoribosylanthranilate isomerase	135	E				194	49	21	2.7
Iho_0963	TBP-interacting protein TIP49	1224	K				450	58	24	2.6
Iho_0542	NADH dehydrogenase subunit C/D	649	C C				540	71	29	2.6
Iho_0759	protein with unknown function			Yes	Yes		202	142	58	2.6
Iho_1413	SSU ribosomal protein S8P	96	J				133	96	38	2.5
Iho_0635	argininosuccinate synthase	137	E				396	106	41	2.4
Iho_1266	Outer membrane pore protein					Yes	85	335	121	2.3
Iho_0981	protein with unknown function			Yes	Yes		1051	73	26	2.3
Iho_1367	Ni-dependent hydrogenase, large sub.	374	C				664	938	337	2.3
Iho_0747	methionine synthase (B12-independent)	620	E				341	473	165	2.2
Iho_0679	Sodium-transporting two-sector ATPase	1156	C				471	1008	348	2.2
Iho_0524	protein with unknown function						159	103	36	2.2
Iho_0590	SSU ribosomal protein S3P	92	J				241	159	54	2.2
Iho_0157	Small nuclear ribonucleoprotein, LSM family	1958	K				91	164	55	2.1
Iho_0363	D-fructose 1,6-bisphosphatase	1980	G				387	1390	465	2.1
Iho_1435	aspartyl-tRNA synthetase	17	J				453	122	40	2.1
Iho_1243	protein with unknown function						182	282	93	2.1
Iho_1305	H+-transporting two-sector ATPase, alpha/beta subunit	1155	C				596	687	220	2.0

The enrichment factor, φ, was calculated as the ratio of the abundance of an individual *I. hospitalis* protein (P) relative to the total *I. hospitalis* proteome (T) between the purified *N. equitans* (N) and the co-culture (I_N) sample (φ = [P_N_/T_N_]/[P_I_N_/T_I_N_]). Total proteome values used in the calculation are T_N_ = 11878, T_I_N_ = 75139.

In an attempt to distinguish between these alternatives we compared the relative abundance of the detected proteins between the *N. equitans* sample and the *I. hospitalis-N. equitans* co-culture, with a general assumption that the more abundant an *I. hospitalis* protein is in the host, the more likely that protein could end up as a significant contaminant in the *N. equitans* fraction. Also, since *N. equitans'* genome encodes all its necessary genetic information processing systems (replication/repair, transcription, translation, and protein folding), proteins involved in those processes are expected not to be transferred. When the detected *I. hospitalis* proteins were grouped based on arCOG categories, no changes in relative abundance were observed between the represented functional categories relative to total proteome of the *I. hospitalis-N. equitans* co-culture. *I. hospitalis* proteins involved in biosynthetic metabolic functions, prominently expressed in both the pure culture and co-culture, were also detected at very low levels in the purified *N. equitans* sample. Collectively, these indicate that no specific enrichment of a broad functional category of *I. hospitalis* proteins occurred in the *N. equitans* sample and that the bulk of the *I. hospitalis* proteins represent contaminants. Therefore, these analyses do not support a hypothesis of a broad transfer of metabolic enzymes from the host to *N. equitans*.

**Figure 6:**
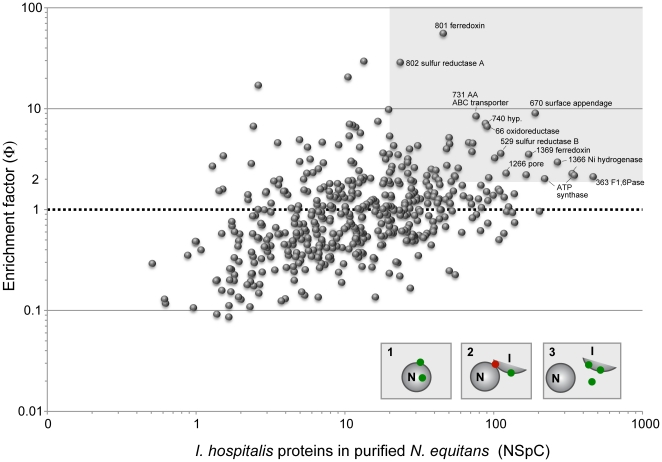
*I. hospitalis* proteins detected in the purified *N. equitans* sample. The graph shows the normalized spectral count of detected proteins versus the enrichment factor, φ. The enrichment factor takes into account the difference in proteome abundance and complexity between the samples. A value of φ = 1 corresponds to no enrichment and can explain protein presence based on carry over/contamination. A region with φ>2 and NSpC>20 was shadowed as comprising of the most likely proteins transfer candidates or otherwise specifically associated with Nanoarchaeum. Below NSpC of 20 the low abundance may affect accurate measurements and those proteins were not taken into account. The insert cartoons depict possible explanations of detecting *Ignicoccus* proteins (green dots) in the Nanoarchaeum sample: (1) real inter-species transfer; (2) adherence of *Ignicoccus* membrane fragments to *Nanoarchaeum*, potentially mediated by a distinct “interactome” (red) or (3) sample contamination with *Ignicoccus* cellular debris.

There remains, however, the possibility that a few select *I. hospitalis* proteins may be incorporated by *N. equitans* either in its membrane or in the cytoplasm. To evaluate this possibility, the individual *I. hospitalis* proteins detected in *N. equitans* were analyzed in terms of both abundance and their “enrichment factor”. We defined that factor, φ, as the ratio of the abundance of an individual *Ignicoccus* protein (P) relative to the total *I. hospitalis* proteome (T) between the purified *N. equitans* (N) and the co-culture (N) samples (φ = [P_N_/T_N_]/[P_C_/T_C_]). A ratio around one indicates no significant enrichment in the *N. equitans* sample and, therefore, the presence of that protein is likely a result of contamination with *I. hospitalis* cellular contents. A higher value (φ>2) may indicate enrichment, potentially as a result of actual transfer from *I. hospitalis* to *N. equitans*. Caveats to this methodology still remain, however. For very low abundance proteins, statistical fluctuations in gene expression and protein measurement may result in high variability. Therefore, proteins for which the normalized spectral counts were below 20 were not taken into account. Less than 50 proteins satisfied these two filtering criteria (φ>2, RSpC>20)(**Table 5** **,** **Figure 6**). Over half of these proteins are predicted to be part of membrane complexes, including subunits of the ATP synthase, Ni hydrogenase, polysulfide reductase and its associated ferredoxin, ABC transporters and the *I. hospitalis* outer-membrane pore oligomer. While these are clearly important components of *I. hospitalis'* energetic metabolism and molecular transport, the question of whether their presence in purified *N. equitans* cells indicates transfer to *N. equitans* or the presence of co-purifying *I. hospitalis* membrane fragments cannot be answered at this time. Similarly, a few other membrane-associated proteins were detected at significant levels, including the recently described appendage protein Iho0670 [14] and two potential cell surface proteins with no known function (Iho0759, 0981). Further biochemical and cellular localization studies will be required in order to map their location in the *I. hospitalis-N. equitans* system and to test whether or not they play a role in the cellular interaction between the two Archaea.

### Conclusions

The relationship between *I. hospitalis* and *N. equitans* remains one of the most intriguing associations in nature, enabled by mechanisms that are still largely elusive. The deep proteomic coverage obtained for both species confirmed the streamlined nature of their genomes, with most genes constitutively expressed and containing relatively few pseudo- or redundant genes. The proteomic study describe here uncovers several of the physiological changes that occur in *I. hospitalis* when *N. equitans* colonizes its surface. A somewhat surprising finding was the relatively low level of proteomic perturbation induced by *N. equitans* on its host's proteome, with only approximately 10% of the proteins exhibiting an increase or decrease in relative abundance by two or more fold. On the other hand, as most *I. hospitalis* genes are expressed under normal conditions and given its strict chemoautotrophic growth with few metabolic alternatives available, perhaps its association with *N. equitans* could only be viable with a tight, selective response at the level of specific genes and processes. The biological effects of small quantitative changes in specific proteins are difficult to predict since different metabolic reactions and cellular processes are controlled by a myriad of mechanisms and rate-limiting reactions. Nevertheless, concerted changes in the abundance of proteins that are co-expressed from the same operons or from different chromosomal regions but are part of the same molecular complexes or metabolic pathways suggests that's changes in some cellular processes may indeed occur.

Observed increases in the abundance of subunits of membrane complexes involved in respiration and ATP synthesis (hydrogenase, sulfur reductase, ATP synthase) suggest an increased energetic and metabolic demand imposed by the presence of *N. equitans*. Since it is not yet possible to measure the respiration and ATP synthesis rates in this system, this remains unverified. At the metabolic level, the co-culture appeared to induce an increase in the abundance of several key enzymes involved in carbon fixation, most notably the pyruvate ferredoxin oxidoreductase, PEP synthase and PEP carboxylase. As every biosynthetic reaction employed by *I. hospitalis* (including all the metabolites that *N. equitans* receives from its host) depend on the reactions catalyzed by those enzymes, this response further substantiates the claim that the association imposes an increased metabolic and energetic demand on the host, *I. hospitalis*.

Other systematic affects induced by *I. hospitalis'* association with *N. equitans* include a reduction of the basal transcriptional machinery and most of the transcriptional regulatory factors. This may result in a general decrease in the overall rate of transcription. Noted, however, was an observed increase in the level of several transcription factors. While the expectation is that these regulatory proteins will specifically influence the expression of selected genes, actual data linking them to either the transcription of mRNA or translation to protein is lacking. Complementing the transcriptional response denoted above, a decrease in the abundance of key translational control proteins (e.g. the translation initiation factor 1A) as well as several proteins involved in protein folding seems to indicate that host cellular growth may be reduced at the expense of increased metabolism in order to support *N. equitans*. This conclusion is similar to microbiological data that indicate a much lower rate of *I. hospitalis* cellular division after an increasing number of *N. equitans* cells have populated their cell surface [2]. These data perhaps converge to a conclusion that *N. equitans* acts much more like a parasite than a commensal, at least under these laboratory culture conditions. Ongoing research focuses on the dynamics of these two organisms, with measurements acquired during progression of a co-culture; a study that incorporates both quantitative proteomics and transcriptomics to garner an enhanced understanding of this relationship.

The proteomic characterization of the purified *N. equitans* sample, while complicated by the potential co-purification of *I. hospitalis* proteins and/or cellular debris, indicates that *N. equitans* does not receive from its host a significant amount (if any) of enzymes that would enable it to perform primary biosynthetic reactions. The mechanism employed by *N. equitans* to acquire amino acids, lipids, nucleotides, still remains unknown. Based on enrichment of specific *I. hospitalis* membrane-associated proteins in the *N. equitans* sample, the potential transfer of complexes involved in generation and maintenance of a membrane potential and ATP synthesis cannot yet be ruled out and will thus require cellular localization studies before a definitive conclusion can be drawn. Several proteins potentially involved in the interaction or molecular transfers between the two organisms emerged and represent targets for further analyses. A combination of systems biology approaches with biochemical and ultrastructural studies are clearly required in order to fully comprehend this fascinating association.

## Materials and Methods

### Cell cultures

*I. hospitalis* and *I. hospitalis-N. equitans* were cultured at 90°C in a 50 liters enamel-protected fermentor using ½ SME medium, elemental sulfur and a H_2_/CO_2_ gas phase, as previously described [2], [32]. The cultures were harvested in stationary phase, rapidly cooled and cells isolated by centrifugation followed by freezing in liquid nitrogen and storage at −80°C. In the *I. hospitalis-N. equitans* co-culture, essentially all *Ignicoccus* harbored multiple *N. equitans* cells on their surface as determined by microscopic analysis [2]. To obtain higher proteome coverage, we also purified an *N. equitans* fraction from a separate co-culture using differential centrifugation [1]. Based on FISH analysis and quantitative PCR with species-specific primers, the purified fraction contained >99% *N. equitans* cells.

### Sample preparation for 2D-LC-MS/MS

Frozen cell pellets (10–50 mg) were re-suspended in 1 ml SDS lysis buffer (4% SDS, 100 mM Tris-HCl pH 8.0, 50 mM DTT) and incubated in boiling water for 5 min. To aid lysis and to completely solubilize the cells, samples were pulse-sonicated (10 s on, 10 s off) for 2 min with an ultrasonic disruptor (Branson) at 20% amplitude. Samples were then boiled again for 5 min, cleared by centrifugation (10 minutes at 21,000 g) and immediately precipitated with 20% trichloroacetic acid (TCA) overnight at −20°C. TCA-precipitated proteins were washed with two additions of ice-cold acetone, air dried, and re-suspended in 8 M urea, 100 mM Tris-HCl pH 8.0. To aid in re-solubilization, samples were sonicated as before, taking care to keep the sample below 37°C, and incubated at room temperature (RT) for 30 minutes. After measuring protein concentration via a BCA assay, samples were adjusted to 10 mM DTT (10 minutes, RT) and then 10 mM iodoacetamide (10 minutes at RT in the dark) to block reduced cysteine residues. Sample aliquots containing ∼1–2 mg of crude protein were diluted to 4 M urea (1∶1) with 100 mM Tris-HCl pH 8.0, 20 mM CaCl_2_, and pre-digested with sequencing-grade trypsin (Promega) at a 1∶75 (w/w) enzyme∶protein ratio overnight at room temperature. Samples were then diluted to 2 M urea (1∶1) and digested with a second aliquot of trypsin (1∶75) for an additional 4 hours. Following digestion, each sample was adjusted to 150 mM NaCl, 0.1% formic acid and filtered through a 10 kDa cutoff spin column filter (VWR brand). The peptide-enriched flow through was then quantified by BCA assay, aliquoted, and stored at −80°C until analysis.

### Measurement of peptides by 2D-LC-MS/MS

For each sample, 50 ug of peptides were bomb-loaded onto a biphasic MudPIT back column [33] packed with ∼5 cm strong cation exchange (SCX) resin for charge-based separation of peptides followed by ∼3 cm C18 reversed phase (RP) for online desalting (Luna and Aqua respectively, Phenomenex). Once loaded, the sample columns were washed offline with solvent A (5% acetonitrile, 95% HPLC-grade water, 0.1% formic acid) for 15 minutes to remove residual urea and NaCl followed by a gradient to 100% solvent B (70% acetonitrile, 30% HPLC-grade water, 0.1% formic acid) over 30 minutes to move the peptide population from the RP to the SCX resin. Washed samples were then placed in-line with an in-house pulled nanospray emitter (100 micron ID) packed with 15 cm of C18 RP material and analyzed via 24-hr MudPIT 2D-LC-MS/MS (11 salt-pulses: 5, 7.5, 10, 12.5, 15, 17.5, 20, 25, 35, 50, 100% of 500 mM ammonium acetate followed by a 100 minute gradient to 50% solvent B) with a hybrid LTQ XL/Orbitrap mass spectrometer (Thermo Fisher) operating in data-dependent mode. Full MS1 scans (2 microscans; 5 MS/MS per MS1) were obtained using an Orbitrap mass analyzer set to 30K resolution, while MS/MS scans (2 microscans) were obtained/performed in the LTQ. A total of three replicate measurements were obtained for each sample.

### Database searching and protein identification

Peptide fragmentation spectra obtained from each of the 6 MS sample measurements were assigned peptide sequences with SEQUEST [34] using a composite FASTA database containing the proteomes of *I. hospitalis*, *N. equitans* (GenBank CP000816.1, AE017199.1), known protein contaminants, as well as reversed entries for all the aforementioned constituents which were used to assess false-discovery rates. In addition, sample processing-induced modifications were taken into account, specifically carboxymethylation of cysteine. Following database searching, DTASelect [35] was used to both filter the data (XCorr: +1 = 1.8, +2 = 2.5, +3 = 3.5, DeltCN 0.08) and consolidate the identified peptides into protein loci. In order for a protein call to be made, it must have been identified by at least two peptides, with one unique to that specific protein entry.

### Data analysis

To prepare for semi-quantitative proteome analysis, DTASelect-filtered data was subjected to spectral count balancing and subsequent NSAF (Normalized Spectral Abundance Factor) determination [36], [37], [38]. With regards to the former, the unique-status of each identified peptide was assessed. If a peptide was deemed unique, it retained 100% of its previously assessed spectral count (SpC). However, if a peptide was found to belong to more than one representative protein, i.e. non-unique, its SpC was recalculated based on the ratio of uniquely identified peptides between the 2 or more proteins that share the non-unique peptide in question. The adjusted SpC of the proteins (aSpC) incorporate these balanced values, which correct for the slight quantitative bias that occurs with homologous proteins and/or proteins with homologous regions, both of which artificially inflate SpC values at the expense of proteins that share no homology. Once spectrally balanced, NSAF values were calculated for each protein in a specific run to normalize individual MS runs based on the total number of spectra collected and protein length, which by itself introduces a bias that favors larger proteins if not corrected for [36]. NSAF values were then multiplied by 46541, the average total spectra count observed across all sample sets. This converts the NSAF decimal value to a theoretical, normalized spectral count (nSpC) value, which is easier to visualize.

As the quantitative focus of this study was to determine how and to what extent the *I. hospitalis* proteome changes in response to *N. equitans*, nSpC values for only *I. hospitalis* proteins were re-normalized so that the total nSpC of the co-culture equaled that of the isolate. These re-normalized nSpC values, denoted as RSpC, were then used in the ensuing analysis. This re-normalization effectively corrects for the observed systematic depression of collected spectra for *I. hospitalis* proteins identified in the co-culture, which was a result of the increased proteomic complexity introduced by the addition of *N. equitans* proteins. Semi-quantitative, RSpC data was then binned into two categories: (i) *I. hospitalis* proteins that were identified in every MS run (3×*I. hospitalis* runs vs. 3×co-culture runs) and (ii) proteins that did not have a complete measurement series. The former category, which by default infers a greater confidence of identification, was used to compute the statistical relevance of each protein's abundance change due to the association of the two organisms. Using standard proteomic data processing [36], [37], the RSpC values were log_2_-transformed to create a normalized distribution of abundance values from which further statistical testing was performed. In this regard, significant changes in abundance for each identified *I. hospitalis* protein, dependent on sample type (pure culture vs. co-culture) was assessed by a Student's T test (p≤0.05).

### Genomic and metabolic inferences

To analyze the proteomic data in a biological context, previous metabolic reconstructions of *I. hospitalis* and *N. equitans* [4], [15] were used and updated based on more recent advances in understanding this archaeal system [19]. Inferred biochemical activities/biological functions used PFAM, KEGG and arCOG [13] classification. Cellular localization was inferred based on Signal P and TMHHM as part of the annotation deposited in IMG [39] as well as based on Pred-Signal, an algorithm optimized for Archaea [40] The role and cellular localization of several proteins has been validated previously [2], [7], [20], [41]. To visualize the relative abundance of the proteins in both genomic and metabolic context, the “Pathways Tools Omics Viewer” from BioCyc was utilized [42], [43]. Operon prediction relied on both BioCyc and DOOR [44]. To identify significant variation in the protein levels between the two culture types and also link them to *I. hospitalis* individual genes and operon structure, we used a Gene Set Enrichment Analysis, GSEA [24]. The *Ignicoccus* proteins were ranked according to their signal-to-noise ratio (the difference of means between NSAF values in both conditions scaled by the standard deviation), then the normalized enrichment score (NES) was calculated as described by Subramanian et al. [24].

## Supporting Information

Figure S1 Frequency distribution of *I. hospitalis* proteins before (NSpC) (**A**) and after balancing (RSpC) (**B**) correcting for the increased proteome complexity in the co-culture with *N.equitans*.
(TIF)

Figure S2 Normalized Enrichment Scores (NES) of the *I. hospitalis* arCOGs calculated by GSEA. The positive scores show the degree of arCOG enrichment the co-culture. The negative scores show the degree of arCOG enrichment in *I. hospitalis* pure culture. Categories marked with * are the most significantly affected (p<0.066).
(TIF)

Table S1 Ignicoccus hospitalis and Nanoarchaeum equitans proteins identified by proteomics and their relative abundance levels. The genes/proteins are arranged in order of gene number based on Genbank. Classifications based on arCOG and membrane association inferences are also shown. For each sample type (*Ignicoccus hospitalis* culture, *Ignicoccus-Nanoarchaeum* co-culture or purified *Nanoarchaeum equitans* sample the sum of the three independent experimental measurements of relative abundances are provided in order of the normalization process: ASpC (Adjusted Spectral Counts); nSpC (Normalized Spectral Counts) RSpC (Re-normalized Spectral Counts). RSpC values were used for all analyses, the other values are only provided as reference.
(XLS)

Supporting Information S1 (DOCX)
