# Supplementary figures and images for: Proteomic Characterization of Cellular and Molecular Processes that Enable the Nanoarchaeum equitans-Ignicoccus hospitalis Relationship
Source: PLoS One. 2011 Aug 3;6(8):e22942. doi: 10.1371/journal.pone.0022942 (PMC3149612; doi:10.1371/journal.pone.0022942)

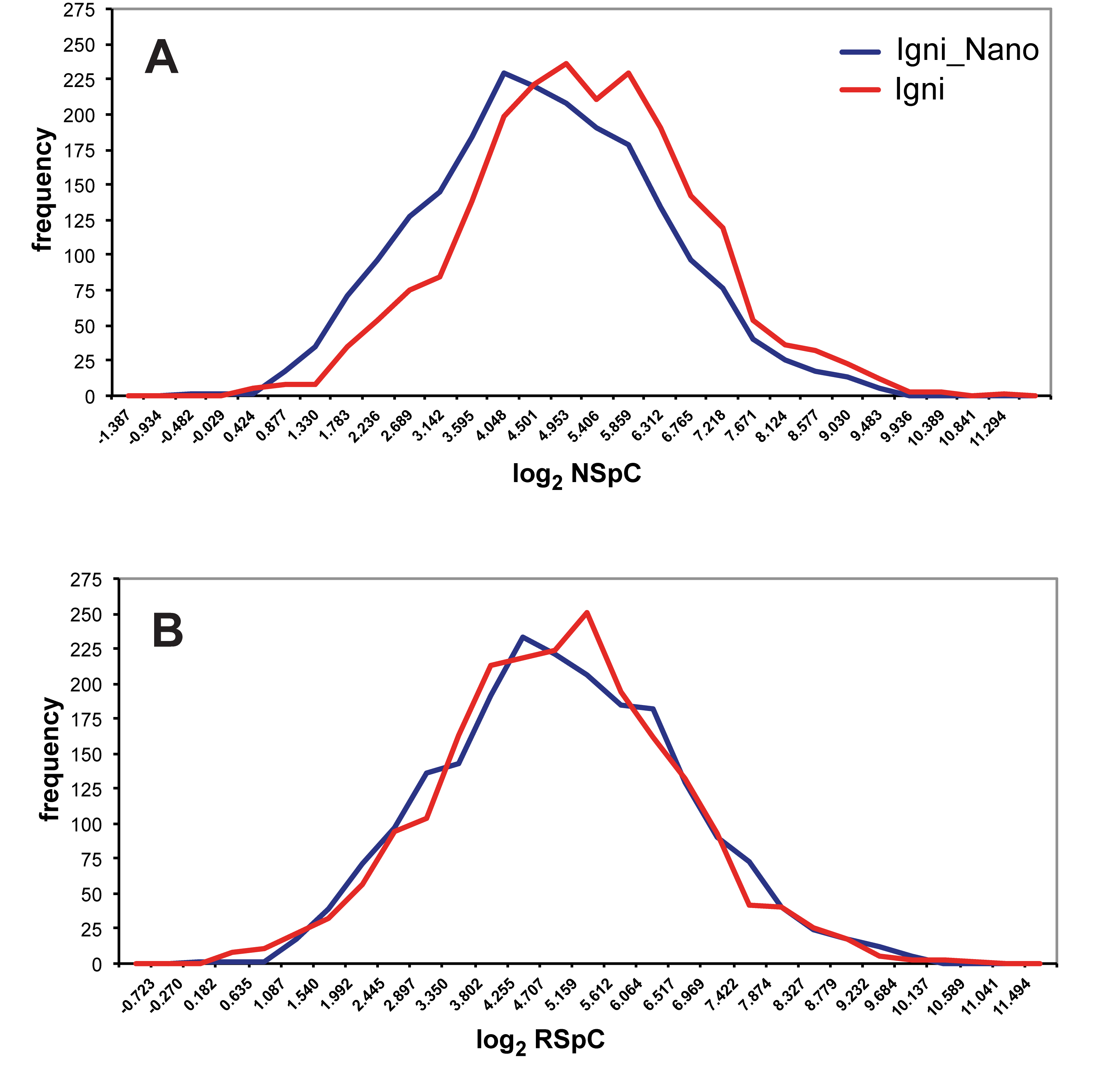

Supplement: Figure S1 — Frequency distribution of I. hospitalis proteins before (NSpC) (A) and after balancing (RSpC) (B) correcting for the increased proteome complexity in the co-culture with N.equitans. (TIF) [file pone.0022942.s001.tif]

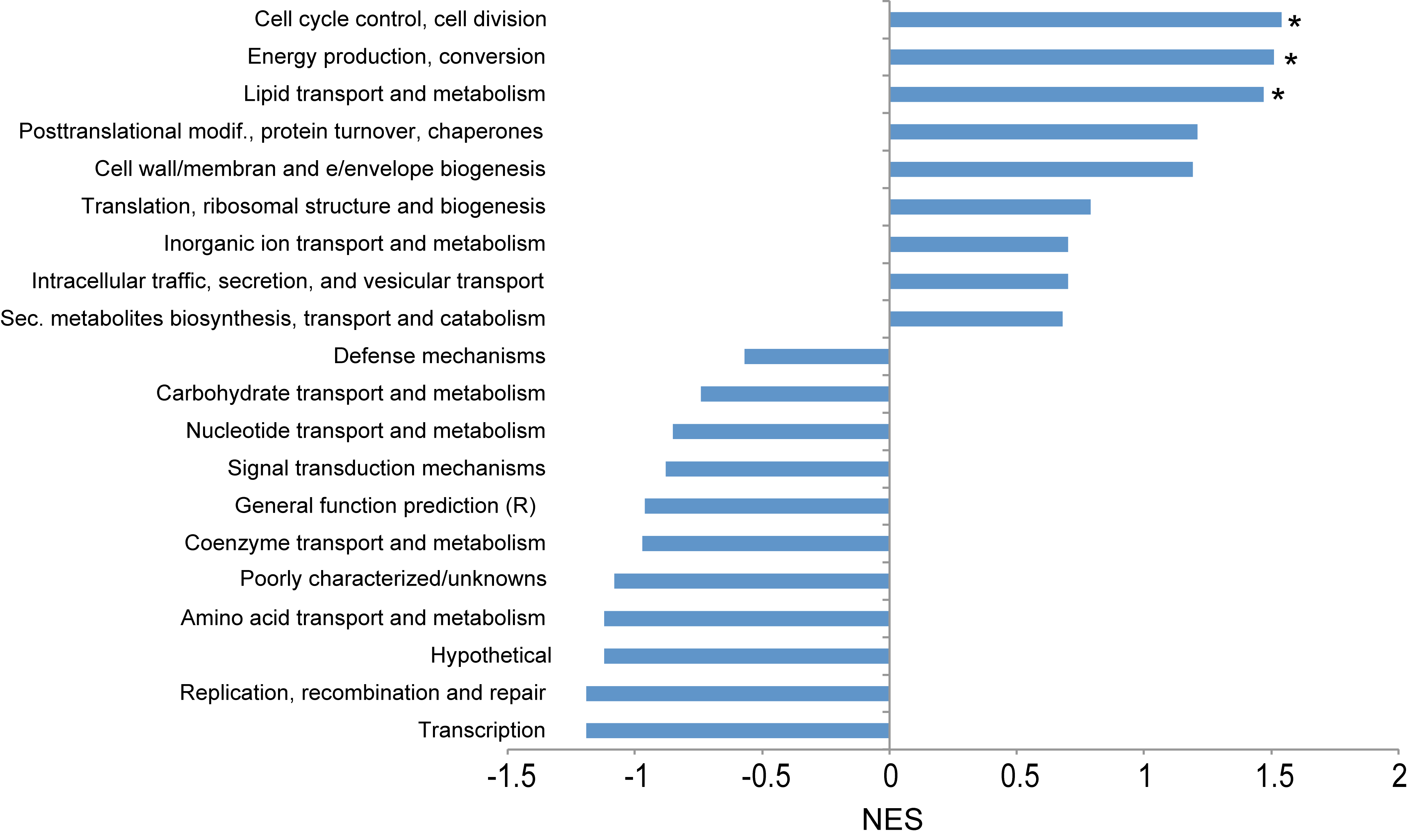

Supplement: Figure S2 — Normalized Enrichment Scores (NES) of the I. hospitalis arCOGs calculated by GSEA. The positive scores show the degree of arCOG enrichment the co-culture. The negative scores show the degree of arCOG enrichment in I. hospitalis pure culture. Categories marked with * are the most significantly affected (p<0.066). (TIF) [file pone.0022942.s002.tif]
