# Supplementary material for: Proteomic Characterization of Cellular and Molecular Processes that Enable the Nanoarchaeum equitans-Ignicoccus hospitalis Relationship
Source: PLoS One. 2011 Aug 3;6(8):e22942. doi: 10.1371/journal.pone.0022942 (PMC3149612; doi:10.1371/journal.pone.0022942)
Supplement: Supporting Information S1 — (DOCX) [file pone.0022942.s004.docx]

**Supporting information**

**Data preparation for semi-quantification comparisons.** Though a goal of this study was to obtain deep proteome measurements for both organisms, as isolates and together in co-culture, the major focus was to determine how and to what extent *I. hospitalis’* proteome changes to accommodate *N. equitans.* In this regard, NSAF values for each identified *I. hospitalis* protein were compared between two conditions, *I. hospitalis* grown as a pure culture versus co-culture with *N. equitans*. Though a similar comparison was performed between the purified *N. equitans* sample and the *N. equitans* component of the co-culture, the fact that the “pure” culture was in fact derived from a co-culture makes this analysis somewhat more difficult to dissect. Comparing proteomes between an isolated organism and a composite of two different organisms presents a unique challenge with regard to semi-quantification by normalized spectra counts. As the proteomic complexity of the co-culture is naturally augmented by the presence of two organisms, the spectral count and thus the log_2_-transformed nSpC value distribution of all identified proteins per organism is systematically depressed (**Supplementary Figure S1a**). In order to compensate for this depression, the nSpC values for each respective analysis (single species versus the co-culture) were renormalized to consider only *I. hospitalis* proteins or *N. equitans* proteins, depending on the desired comparative analysis (**Supplementary Figure S1b**). Once corrected, proteins that were identified in all respective MS runs (i.e abundance of *I. hospitalis* proteins in the pure culture versus the co-culture) could be compared without the observed bias. Thus**,** the rebalanced nSpC values (RSpC) incorporate normalization on several levels: (i) protein length, (ii) total spectra collected, and (iii) proteomic complexity.

RSpC values, like raw SpC values (see above) cover three orders of magnitude with regard to the relative abundance of each protein. Considering just *I. hospitalis* proteins identified in all replicates across both the pure and co-cultures (706 total proteins), RSpC values ranged from 1.1 to 1884.6. These values, though respectable, are effectively shortchanged due to the conservative peptide filters placed on the SEQUEST-searched data prior to normalization and rebalancing. More specifically, the required “2-peptide minimum” for a protein call eliminates so-called “one-hit wonders” at the raw SpC-level and thus reduces the magnitude of the dynamic range. Although the absence of mass standards precludes the precise determination of the actual concentration of individual proteins in the cell, it has been shown that proteomic normalization methodologies such as NSAF are effective in estimating protein abundance trends despite some of the known systematic biases inherent to mass spectrometry-based shotgun proteomic measurements [1]. In addition, semi-quantitative measurements are relative by nature and thus most bias is factored out when comparing a protein to itself between samples and conditions.

1. Zybailov B, Mosley AL, Sardiu ME, Coleman MK, Florens L, et al. (2006) Statistical

analysis of membrane proteome expression changes in *Saccharomyces cerevisiae*. J

Proteome Res 5: 2339-2347.
